# A possible case of caprine-associated malignant catarrhal fever in a domestic water buffalo *(Bubalus bubalis) *in Switzerland

**DOI:** 10.1186/1746-6148-7-78

**Published:** 2011-12-02

**Authors:** Martina Dettwiler, Anina Stahel, Sonka Krüger, Christian Gerspach, Ueli Braun, Monika Engels, Monika Hilbe

**Affiliations:** 1 Institute of Veterinary Pathology, Vetsuisse Faculty, University of Zurich, Winterthurerstrasse 268, CH-8057 Zurich, Switzerland; 2 Institute of Veterinary Virology, Vetsuisse Faculty, University of Zurich, Winterthurerstrasse 266a, CH-8057 Zurich, Switzerland; 3 Department of Farm Animals, Vetsuisse Faculty, University of Zurich, Winterthurerstrasse 260, CH-8057 Zurich, Switzerland

## Abstract

**Background:**

Malignant catarrhal fever (MCF) is a fatal herpesvirus infection, affecting various wild and domestic ruminants all over the world. Water buffaloes were reported to be particularly susceptible for the ovine herpesvirus-2 (OvHV-2) causing the sheep-associated form of MCF (SA-MCF). This report describes the first case of possibly caprine-associated malignant catarrhal fever symptoms in a domestic water buffalo in Switzerland.

**Case presentation:**

The buffalo cow presented with persistent fever, dyspnoea, nasal bleeding and haematuria. Despite symptomatic therapy, the buffalo died and was submitted to post mortem examination. Major findings were an abomasal ulceration, a mild haemorrhagic cystitis and multifocal haemorrhages on the epicardium and on serosal and mucosal surfaces. Eyes and oral cavity were not affected. Histopathology revealed a mild to moderate lymphohistiocytic vasculitis limited to the brain and the urinary bladder. Although these findings are typical for MCF, OvHV-2 DNA was not detected in peripheral blood lymphocytes or in paraffin-embedded brain, using an OvHV-2 specific real time PCR. With the aid of a panherpesvirus PCR, a caprine herpesvirus-2 (CpHV-2) sequence could be amplified from both samples.

**Conclusions:**

To our knowledge, this is the first report of malignant catarrhal fever in the subfamily *Bovinae*, where the presence of CpHV-2 could be demonstrated. The etiological context has yet to be evaluated.

## Background

Malignant catarrhal fever (MCF) is a frequently fatal viral infection, affecting various wild and domestic ruminants and even, as recently reported, pigs [1]. The disease is thought to be caused by herpesviruses of the subfamily *gammaherpesvirinae*, which cluster within the genus *Macavirus *[2]. Typical signs of MCF include fever, nasal and ocular discharge, corneal opacity along with erosive lesions on the muzzle, within the oral cavity as well as in the lower gastrointestinal tract, the latter associated with melena and haematuria. Histologically, a lymphohistiocytic vasculitis, sometimes accompanied by fibrinoid necrosis, is present in various organs in most cases [3]. Affected animals normally die, with survivors remaining chronically infected, often displaying persistent eye lesions and arteriopathy [4]. Emaciation and dermal lesions occurring in deer infected with these herpesviruses can also be considered a chronic form of MCF [5-7].

At least six members of the *Macaviruses *are associated with clinical MCF [8]. The first identified MCF virus was the alcelaphine herpesvirus-1 (AlHV-1) [9], which persists as a subclinical infection in wildebeest *(Connochaetes sp.) *and is the causative agent of wildebeest-associated MCF (WA-MCF) or African MCF in susceptible ruminants [10]. The ovine herpesvirus-2 (OvHV-2) is endemic in domestic sheep *(Ovis aries) *worldwide and causes sheep-associated MCF (SA-MCF), reported in domestic cattle [3,4,11], bison *(Bison bison) *[12], water buffalos *(Bubalus bubalis) *[11,13,14], various deer species [15-17] and swine *(Sus scrofa domesticus) *[1].

Li and colleagues found a new endemic and asymptomatic gammaherpesvirus in domestic goats *(Capra hircus)*, named caprine herpesvirus-2 (CpHV-2) [18]. This virus can cause clinical MCF in several deer species such sika deer *(Cervus nippon) *[5,6,17], roe deer *(Capreolus capreolus)*, moose *(Alces alces) *[15] and white-tailed deer *(Odocoileus virginianus) *[7,19].

A further gammaherpesvirus with an as yet unknown host reservoir was recovered from white-tailed deer diagnosed with MCF and was named malignant catarrhal fever virus of white-tailed deer (MCFV-WTD) [19,20].

Alcelaphine herpesvirus-2 (AlHV-2) causes subclinical infection in topi *(Damaliscus korrigum) *[21] and hartebeest *(Alcelaphus buselaphus) *[22]. An AlHV-2-like virus was found as the causative agent of MCF in Barbary red deer *(Cervus elaphus barbarus) *[23]. In blood samples of healthy zoo or wildlife park ruminants, including Nubian ibex *(Capra nubiana)*, gemsbok *(Oryx gazella)*, muskox *(Ovibos moschatus) *and aoudad *(Ammotragus lervia)*, several novel homologous gammaherpesvirus sequences have been detected [24,25]. The viruses were named after their carrier species (MCFV-Ibex, MCFV-Oryx, MCFV-Muskox, MCFV-Aoudad). In 2007, MCFV-Ibex was found in a bongo antelope *(Tragelaphus euryceros) *with MCF [26]. Furthermore, hippotragine herpesvirus-1 (HiHV-1) has been detected in asymptomatic roan antelopes *(Hippotragus equinus) *[27].

This case report describes the clinical, laboratory and postmortem findings of a domestic water buffalo *(Bubalus bubalis) *with MCF in Switzerland. Surprisingly, the buffalo was PCR-negative for OvHV-2, but was positive for caprine herpesviral DNA. To the authors' knowledge, this is the first reported case of malignant catarrhal fever in a member of the subfamily *Bovinae *where the presence of CpHV-2 could be demonstrated.

## Case presentation

### Anamnesis, clinical history and laboratory findings

In January 2011, a 10-year old female domestic water buffalo *(Bubalus bubalis) *was referred to the Department of Farm Animals, University of Zurich, Switzerland, with a two week history of fever and dyspnoea resistant to therapy with antibiotics, nonsteroidal anti-inflammatory drugs and β_2_-sympathomimetics. The animal originated from a subalpine farm in Eastern Switzerland that housed 17 adult and 6 juvenile water buffalos, 13 dairy cows, 6 juvenile cattle, 4 suckling calves, 30 ewes, 2 horses and 12 pigs. During the summer months, the buffalos were kept on pasture with intermittent contact to goats and sheep. Cattle and buffalos all tested negative for IBR-IPV (infectious bovine rhinotracheitis) and BVD-MDV (bovine virus diarrhoea/mucosal disease).

The buffalo cow had delivered a healthy calf in June 2010 and was now pregnant in the first trimester.

On arrival at the clinic the animal was in a good body condition. It showed nasal bleeding from the right nostril and a skin lesion on the left fore limb with prolonged bleeding. The rectal temperature was 38.6°C, the skin was cool and had a reduced turgor. Conjunctivae and sclerae were hyperaemic, capillary refill time was prolonged (> 2 sec). The animal was calm, but had an increased heart rate (104/min). Ruminal as well as intestinal motility were absent, food intake was reduced. The quantitatively diminished faeces contained a marked amount of digested blood (melena). Although the peripheral lymph nodes were inconspicuous, the right iliac lymph node was severely enlarged. Catheter urine was brownish to red and contained a maximal amount of haemoglobin/erythrocytes as well as glucose (*Combur-Test*^®^, Roche Diagnostics, Basel, Switzerland). In the glutaraldehyde test (*Glutal-Test*^®^; Dr. E. Graeub AG, Berne, Switzerland), the blood clotting time was longer than 10 minutes.

The results of routine blood examination and special clotting parameters are listed in table 1. The red blood cell values were at the low end of the normal range, leukocytes (not differentiated) were normal and thrombocytes could not be analysed (clotting). Total bilirubin was moderately increased, urea, aspartate-amino-transferase (ASAT, GOT), sorbitol dehydrogenase (SDH) and creatine kinase (CK) were slightly above normal and electrolytes, except for potassium, were moderately (Na, Cl, Mg) to severely (Ca, P) decreased. All clotting parameters assessed (prothrombin time (PT), partial thromboplastin time (PTT), thrombin clotting time (TCT)) were severely prolonged and fibrinogen was in the lower normal range.

**Table 1:** Blood parameters of the buffalo cow with MCF

Parameter	Unit	Patient's value	Ref. value
Hematocrite	%	27	25-33

Erythrocytes	x10^6^/μl	4.77	4.9-6.9

MCHC	g/dl	32	35-36

Leukocytes	x10^3^/μl	8.2	4.0-8.8

Platelets	x10^3^/μl	NR*	238-509

Total proteins	g/l	58	63-86

Fibrinogen	g/l	2	2-10

Total bilirubin	μmol/l	32.7	1.5-2.9

Urea	mmol/l	7.3	2.4-6.5

ASAT (GOT)	U/l	210	57-103

SDH	U/l	15.8	4.0-7.4

CK	U/l	954	70-169

Sodium	mmol/l	136	144-151

Potassium	mmol/l	4.2	3.9-5.0

Chloride	mmol/l	97	100-108

Calcium	mmol/l	1.96	2.3-2.6

Magnesium	mmol/l	0.70	0.8-1.0

Inorganic phosphate	mmol/l	0.48	1.3-2.4

Prothrombin time (PT)	sec	70.7	19.5-25.7

Partial thromboplastin time (PTT)	sec	71.4	21.3-40.5

Thrombin time (TT)	sec	71.6	18.8-23.9

* No result, thrombocytes were clotted.

Routine serology for IBR-IPV antibody was negative. DNA was extracted from peripheral blood lymphocytes with the *QIAamp DNA Mini Kit*^® ^(Qiagen, Hombrechtikon, Switzerland). The DNA was tested by real-time PCR specific for OvHV-2 essentially as described by Hüssy et al. [28] using the following primers and probe: forward 5'-GAGAACAAGCGCTCCCTACTGA-3', reverse 5'-CGTCAAGCATCTTCATCTCCAG-3', probe FAM-AGTGACTCAGACGATACAGCACGCGACA-TAMRA. No OvHV-2 specific DNA could be detected.

Prolonged clotting parameters, decreased fibrinogen and clotted thrombocytes were suggestive of disseminated intravascular coagulation (DIC) and, as underlying disease, an atypical postparturiant haemoglobinuria or malignant catarrhal fever due to a causative agent other than OvHV-2 was considered. The buffalo cow was treated symptomatically by oral and intravenous administration of electrolytes, glucose and vitamin C.

During the following 36 hours, the rectal temperature rose to 39.4°C. The animal was restless and collapsed several, times resulting in bleeding skin injuries. Nasal bleeding stopped intermittently. A few hours before it died, it became recumbent and shortly before exitus showed severe dyspnoea and acute bleeding arose from nose, vagina and anus.

### Postmortem examination

**Figure 1:**
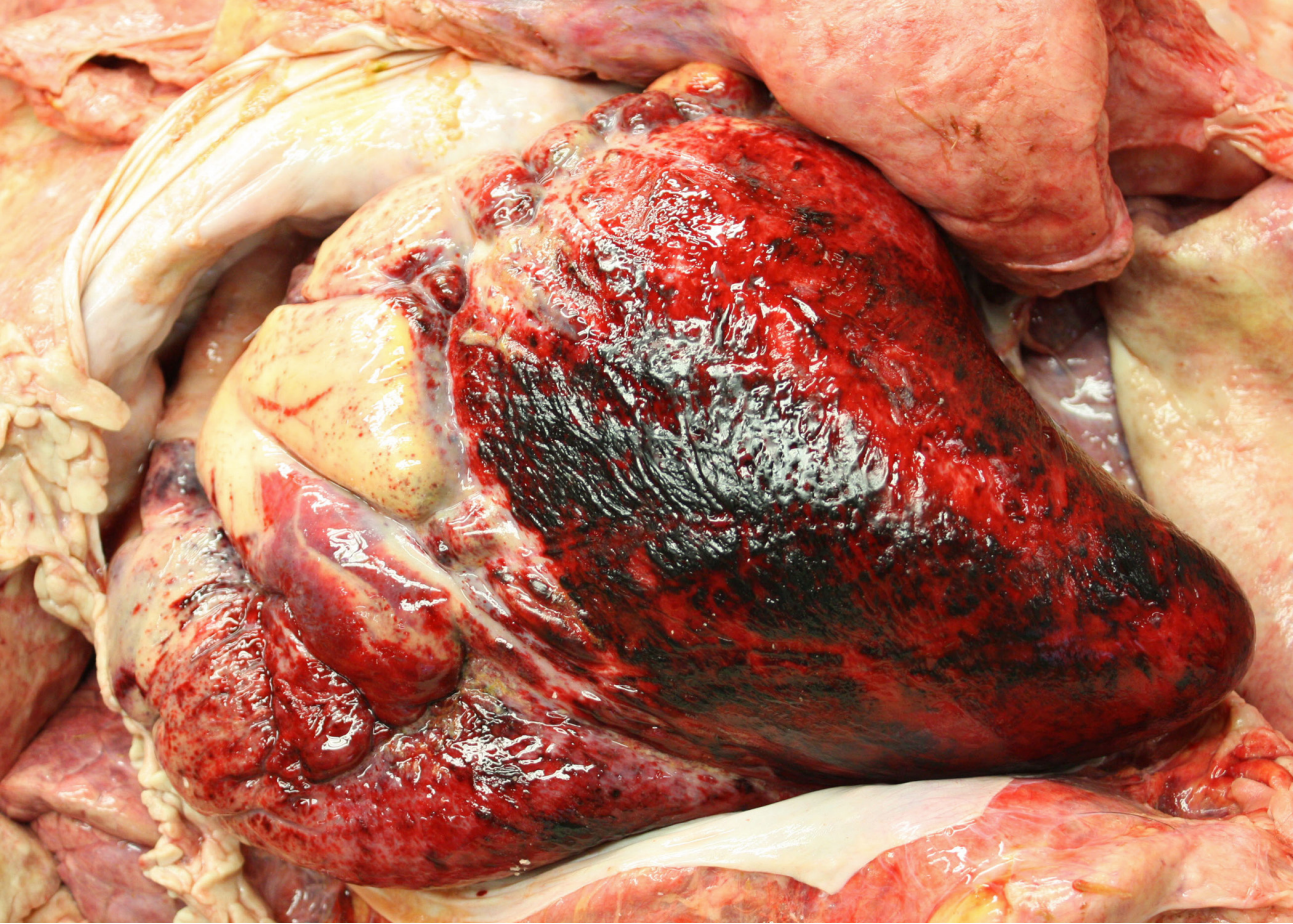
**Epicardial haemorrhages**. Severe multifocal to coalescing epicardial haemorrhages in the water buffalo cow with MCF. The pericardium contained 1,5 liters of serous fluid (evacuated).

The following post mortem examination revealed extensive haemorrhages in the musculature and subcutaneous tissue above the sternum and tuber coxae. Petechial to ecchymotic haemorrhages were seen in the pale vaginal mucosa, in the lung parenchyma of all lobes, in the epicardium (Figure 1) and multifocally on the large omentum, in the abdominal fatty tissue as well as in the mucosa of the urinary bladder. A marked hydropericardium and a mild serous ascites were present.

The tonsils and head lymph nodes were strongly enlarged, red to violet and oedematous. Mediastinal and mesenterial lymph nodes were of normal size, but also dark red and oedematous.

Except for a focally extensive erosion in the pyloric region of the abomasum, no lesions were detectable on the muzzle, in the oral cavity, oesophagus or lower gastrointestinal tract. The eyes showed no macroscopical alterations.

**Figure 2:**
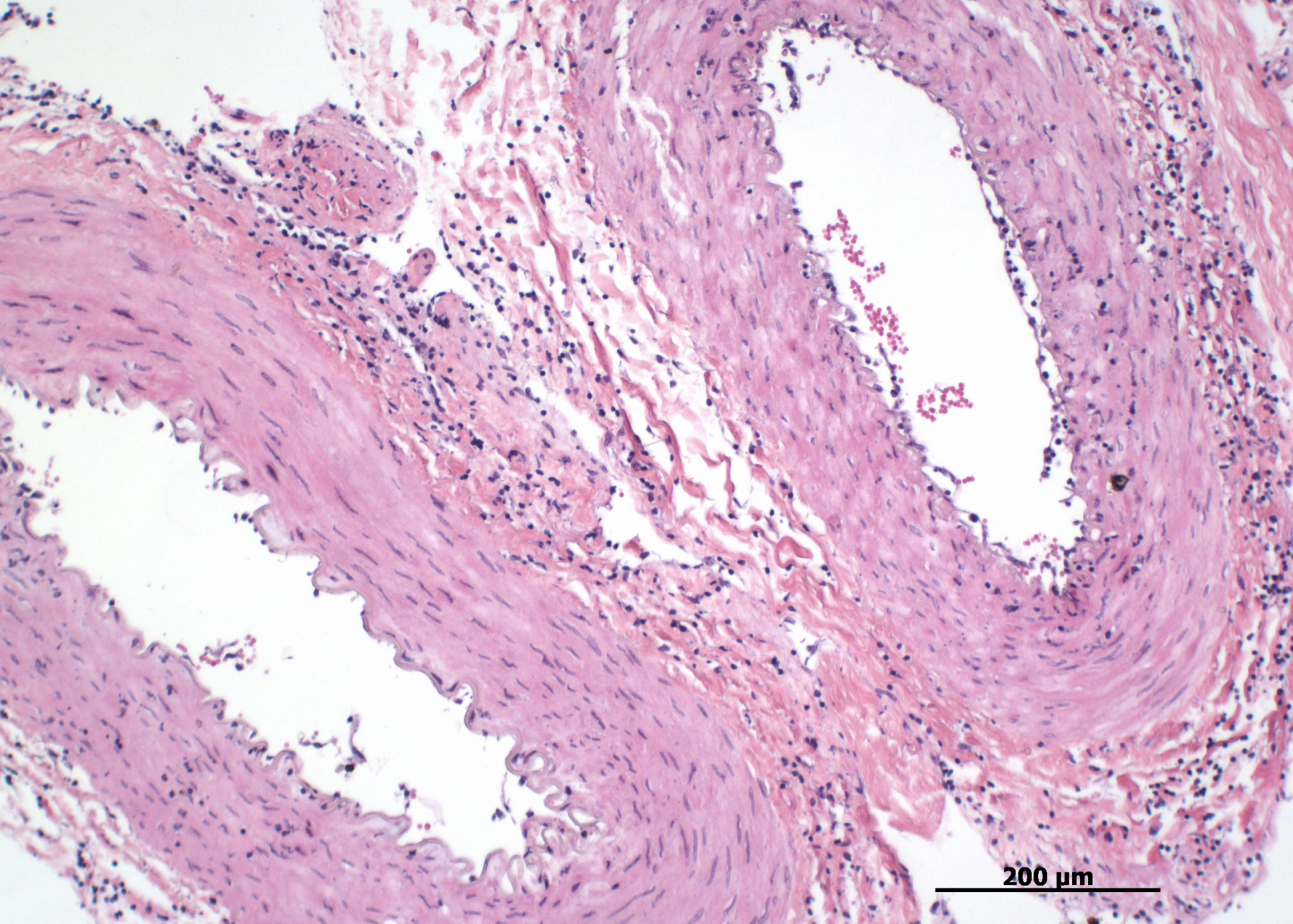
**Lymphohistiocytic vasculitis of the carotid rete**. Lymphohistiocytic medial and adventitial infiltration in arteries of the carotid rete in the water buffalo cow with MCF. Hematoxilin and Eosin, 20×.

The major histological finding was a mild to moderate lymphohistiocytic vasculitis in the brain, in the carotid rete (Figure 2), in the meningeal vessels of the cerebellum and in the urinary bladder wall. The vascular inflammation was mainly characterised by an adventitial infiltration and perivascular cuffing consisting of lymphocytes and histiocytes. In the carotid rete, an additional medial infiltration consisting of mainly lymphocytes and a few neutrophils was seen. Fibrinoid necrosis was not evident in any affected vessel.

Several organs showed signs of bleeding and thrombus formation. Petechial to ecchymotic haemorrhages were dispersed in the submucosa of the urinary bladder, in the epicardium and myocardium, in the cerebrum and in the lung alveoli. Microthrombi were evident in the kidney, in the abomasum adjacent to the mucosal erosion and in the alveolar capillaries of the lung. Multiple lung venules were obstructed by larger thrombi.

Tonsils and lymph nodes were hyperaemic, oedematous and moderately hyperplastic. A severe hemosiderosis was visible in the spleen despite advanced autolysis. In the bone marrow, activation of erythropoiesis and thrombocytopoiesis was evident.

Cryosections from skin, tongue and thyroid gland were examined with immunohistochemistry for BVD-MD antigen [29]. The result was negative. An immunohistochemical examination of formalin-fixed, paraffin-embedded hippocampus and cerebellum for rabies antigen, using a polyclonal antibody, was negative as well.

No relevant bacteria could be cultured from blood.

The OvHV-2-specific real-time PCR was additionally carried out with DNA extracted from paraffin embedded carotid rete and cerebrum (*QIAamp DNA Mini Kit*, Qiagen), which was negative. We then performed a panherpesvirus PCR according to Ehlers et al. [30]. The resulting amplicon was sequenced (Microsynth, Balgach, Switzerland) after gel extraction (*QiaAmp gel extraction kit*; Qiagen) and the sequence was analysed by a BLAST search. The sequence was identical to the corresponding portion of the CpHV-2 polymerase gene.

## Discussion

Buffalo farming is quite uncommon in Switzerland and information on local prevalence of buffalo diseases is sparse. Data from Switzerland concern mainly dairy cattle. To find information about prevalences of buffalo diseases, we can draw conclusions from countries, where buffalos are often kept, like India, Thailand, Indonesia etc., but have to consider that there are differences in housing and feeding, as well as in climate conditions.

The only European report describing an outbreak of sheep-associated MCF in water buffalos occurred in southern Italy [14]. All affected animals were 10-12 months of age and suffered from the "head and eye" form of the disease, characterized by fever, keratoconjunctivitis, oculo-nasal discharge and erosive lesions in the oral cavity and in the laryngo-pharynx. All affected calves had neurological symptoms such as depression and ataxia. In Asian buffalos, however, eye symptoms seemed not to be that severe. Hoffman et al. [31] and Teankam et al. [13] noticed an inconsistent presence of corneal opacity, whereas mild to moderate conjunctivitis was a common symptom. In the present case, the eyes were macroscopically normal and a histologic examination was not performed. As has been described in cattle with SA-MCF [3,4,11], an ulcerative stomatitis and abomasitis, a diphteric tracheitis and/or pharyngitis and a lung oedema can also be found in affected buffalo [13,31]. Further common gross pathological findings in buffalo are petechial or ecchymotic haemorrhage on mucosal and serosal surfaces, pericardial and epicardial haemorrhages, serofibrinous epicarditis and myocarditis and oedematous lymph nodes [13,31]. In the present case, ulceration of the alimentary tract was limited to the abomasal mucosa. Cardial alterations were characterized by a marked hydropericardium and epicardial haemorrhages, most likely due to vascular injury. Other reported findings such as epicarditis, pharyngitis, tracheitis and a lung oedema were absent.

Histologically, the lymphohistiocytic vasculitis and perivascular cuffing was limited to the brain and meningeal vessels, the carotid rete and the urinary bladder wall. Nonpurulent interstitial nephritis and myocarditis, as seen in affected cattle and buffalos [13,31], were not present. In accordance with Hoffman [31], but in contradiction to Teankam [13], fibrinoid necrosis of vessel walls was not evident in any organ examined. Petechial to ecchymotic haemorrhage was distributed multifocally in the myocardium, in the urinary bladder wall, in the white matter of the cerebrum and in the lung alveoli. Especially in the bladder and in the brain, the bleeding was closely associated with inflamed vessels, suggesting a severe vascular injury, whereas in the lung the alveolar haemorrhage can be considered a consequence of circulatory disturbances because of the thrombotic lung venules. This hypercoagulatory state as part of disseminated intravascular coagulation can be explained by the loss of plasma coagulation factors through injured vessel walls and is the most likely cause of death of the buffalo cow.

In summary, the gross and histopathological findings in the present case are consistent with previous descriptions of buffalos succumbing to MCF, albeit in a more discrete manner, although the clinical signs were rather atypical. Possibly, this is related to a different causative agent, as previous cases of MCF in buffalos were associated with OvHV-2, whereas in the present case, CpHV-2 was found in peripheral blood lymphocytes and in brain.

Several authors previously described caprine herpesvirus 2 as a cause of MCF, but exclusively in members of the cervid family. In sika deer, CpHV-2 was found in association with mural folliculitis [5], extensive alopecia, crusting dermatitis and weight loss [6,17]. All of these sika deer had a marked lymphocytic meningoencephalitis and adventitial lymphocytic infiltration in several organs including affected skin, consistent with MCF. CpHV-2 associated MCF in white-tailed deer also manifested in weight loss, alopecia and histologically typical alterations [7]. In free-ranging moose and roe deer, the most prominent clinical symptoms were abnormal behaviour, apathy and incoordination. Macroscopically inconspicuous roe deer displayed vascular and perivascular lymphohistiocytic infiltrates in the eye, in the brain and in multiple organs. A moderate erosive stomatitis and rhinitis respectively, was found in moose, together with lymphohistiocytic infiltrates in several organs, including fibrinoid necrosis in brain vessels [15].

In summary, cervids with CpHV-2 associated MCF presented with rather atypical clinical signs and rather mild to moderate pathological alterations. However, it should be considered, that in most cases these animals did not die a natural death, but were shot by a gamekeeper due to weakness, what might at least partly contribute to reduced severity of the pathological findings.

## Conclusion

As in the case of the water buffalo presented here, CpHV-2 associated MCF elicits rather an atypical clinical and gross pathological manifestation in cervids, suggesting that the caprine herpesvirus 2 causes a more atypical form of MCF in other susceptible species.

Unfortunately, further testing on the premises for MCF viruses or MCF virus antibodies was not permitted by the owner. Therefore, it was impossible to unambiguously identify the source of the infection. Specifically, the question, whether or not CpHV-2 was also present in the unaffected buffaloes on the same premises, remained unanswered. However, clinical, pathological, histological, and virological evidence strongly suggests that CpHV-2 may have been the causative agent of the present case of MCF in a water buffalo. Further knowledge about the prevalence of CpHV-2 in other bovids is needed, raising the need to test MCF-affected cattle for CpHV-2, especially if they are negative for OvHV-2 and contact to goats cannot be excluded. A prevalence study on CpHV-2 among cervids and bovids in Switzerland is in preparation (personal communication, A. Stahel).

## Consent

Consent was obtained from the owner of the animal for publication of this case report and any accompanying images.

## Competing interests

The authors declare that they have no competing interests.

## Authors' contributions

MD performed the necropsy and the histopathological examination, reviewed the literature and prepared the manuscript. AS and ME performed the virological examination. SK and CG carried out the clinical examination, UB supervised the clinical examination. MH supervised the necropsy and the histopathological examination. All authors read and approved the final manuscript.
